# Transcranial doppler as screening method for sickling crises in children with sickle cell anemia: a latin America cohort study

**DOI:** 10.1186/s12887-022-03429-5

**Published:** 2022-06-27

**Authors:** Gabriel Pinheiro Modolo, Gustavo José Luvizutto, Pedro Tadao Hamamoto Filho, Gabriel Pereira Braga, Silmeia Garcia Zanati Bazan, Natalia Cristina Ferreira, Juli Thomaz de Souza, Fernanda Cristina Winckler, Carlos Clayton Macedo de Freitas, Newton Key Hokama, Edison Iglesias de Oliveira Vidal, Rodrigo Bazan

**Affiliations:** 1 Departmento de Neurologia, Psicologia e Psiquiatria, Universidade Estadual Paulista (UNESP), Botucatu, Brasil; 2 Departmento de Fisioterapia Aplicada, Universidade Federal Do Triângulo Mineiro (UFTM), Uberaba, Minas Gerais Brasil; 3 Faculdade de Medicina, Universidade Federal de Mato Grosso Do Sul (UFMS), Campo Grande, Mato Grosso Do Sul Brasil; 4 Departamento de Clínica Médica, Universidade Estadual Paulista (UNESP), Botucatu, Brasil

**Keywords:** Stroke, Sickle cell anemia, Transcranial Doppler

## Abstract

**Background:**

Sickle cell anemia (SCA) is the leading cause of childhood stroke. We aimed to evaluate whether altered cerebral flow velocities, as measured by transcranial Doppler (TCD), are associated with vaso-occlusive complications in addition to stroke in pediatric SCA patients.

**Methods:**

We evaluated 37 children aged between 2 and 16 years with SCA who underwent screening for TCD between January 2012 and October 2018. Genotypic profiles and demographic data were collected, TCD examinations were performed during follow-up, and the presence of sickling crises was compared. Survival analyses were performed using simple frailty models, in which each predictor variable was analyzed separately in relation to the occurrence of a sickling crisis.

**Results:**

The variables related to sickle cell crises in the univariate analysis were peak systolic velocity (PSV) in the middle cerebral artery (MCA), hazard ratio (HR) 1.01 (1.00—1.02) *p* = 0.04; end-diastolic velocity (EDV) in the MCA, HR 1.02 (1.01—1.04) *p* = 0.01; time average mean maximum velocity (TAMMV) in the basilar artery (BA), HR 1.02 (1.00—1.04) *p* = 0.04; hemoglobin, HR 0.49 (0.38—0.65) *p* < 0.001; hematocrit, HR 0.78 (0.71—0.85) *p* < 0.001; leukocyte counts, HR 1.1 (1.05—1.15) *p* < 0.001; platelets counts, HR 0.997 (0.994—0.999) *p* = 0.02; and reticulocyte numbers, HR 1.14 (1.06—1.23) *p* < 0.001.

**Conclusions:**

Our results indicate PSV and EDV in the MCA and TAMMV in the BA as markers of risk for the occurrence of sickling crises in SCA.

**Supplementary Information:**

The online version contains supplementary material available at 10.1186/s12887-022-03429-5.

## Background

Sickle cell anemia (SCA) is caused by abnormal beta-globin alleles that carry a sickle cell mutation within the *HBB* gene (Glu6Val, βS). It is one of the most common monogenic disorders in hematology [1]. Approximately 300,000 children worldwide are born with SCA each year [2]. The main theory for the evolutionary emergence of the disease is that the mutation leading to the sickling of red blood cells protects against the effects of malaria [3]. Demand for health services and hospitalizations among these patients is frequent. In the United States, an estimated one billion dollars is spent annually on SCA [4].

The pathophysiology of SCA is related to structural alterations that occur in sickle shaped red blood cells (HbS). This is a variant of normal adult hemoglobin (HbA) [2]. The clinical manifestations of SCA are multisystemic and begin within the first few months and years of life. SCA involves chronic complications, such as cognitive impairment, pulmonary hypertension, and retinopathy, as well as complications related to disease treatment, such as cirrhosis (due to iron accumulation). Complications are primarily associated with injuries caused by ischemia, hypoxia, and tissue inflammation, leading to acute and chronic involvement of target organs (Additional File 1). These hurdles lead to substantial impairments in patients’ quality of life [5].

Chronic complications of SCA may be related to the pathophysiology and/or treatment of the disease. Pathology is caused by chronic vascular injury resulting from tissue hypoxia-reperfusion, hemolysis, sustained anemia, vascular occlusion, endothelial erythrocyte adhesion, and/or chronic inflammation (Additional File 2) [3]. Children with SCA have a 300-fold increased risk of stroke, making it the most common cause of childhood stroke [6–8]. In the long term, stroke can lead to physical and cognitive disability [3]. The risk of stroke in these patients is related to the time average mean maximum velocity (TAMMV) in the middle cerebral arteries and the intracranial terminal internal carotid artery [8]. Adams et al. used these measures to develop a follow-up protocol with transcranial Doppler (TCD) for patients aged 2—16 years, and indications for blood transfusion aim to reduce HbS and TAMMV. This protocol reduces the risk of stroke by approximately 90% in these patients [8].

The TCD screening protocol proved to be effective and reproducible [9]. The flow rates evaluated by TCD were correlated with severity of anemia [10]. Similarly, low hematocrit and hemoglobin levels were associated with complications of the disease [11]. It is therefore interesting to assume that changes in the velocities identified by TCD examination may reflect the severity of SCA. However, the ability of the TCD examination to predict a higher risk of SCA complications outside the central nervous system, remains to be determined.

Although very efficient in preventing stroke, the TCD screening protocol is poorly implemented, even in resourceful countries. Less than 50% of children in Europe receive annual TCD examinations [12]. In a survey conducted at 28 sites using annual TCD screenings, differences in the stroke prevention practices demonstrated the real-world challenges from translation of trials into clinical practice [13]. However, evaluation of flow velocities in the intracranial arteries by TCD screening is effective for stratifying stroke risk, allowing for prophylactic blood transfusions. Examination results are correlated with the severity of the anemia and vasculopathies resulting from SCA. Estepp et al. demonstrated that an increase in Hb concentration is a strong influencer of TAMMV reduction [14]. Hemolysis indicators, such as reticulocyte counts and bilirubin levels, correlate significantly to TCD velocities [15]. However, any relationship between flow velocities and adverse events outside the nervous system has not been well established.

In view of these questions, the objectives of this study were: 1) to evaluate and describe the relationship between the flow velocities measured by TCD and the occurrence of sickling crises and 2) to evaluate the association between the presence of abnormal TCD examination results and occurrence of vaso-occlusive syndromes. The main hypothesis stated that assessment of flow velocities in the intracranial arteries using TCD may predict an increased risk of sickling crises.

## Methods

### Study design, setting, and participants

This was a retrospective cohort study of patients with a genetic diagnosis of SCA, aged between 2 and 16 years, who visited the hematology outpatient clinic of Botucatu Medical School (UNESP) from January 2012—October 2019. All patients between 2 and 16 years of age have been undergoing screening with TCD since 2010 at the neurosonology clinic, under the direction of the vascular neurology services. Patients underwent blood transfusion treatments according to international recommendations [16]. There is a specific outpatient clinic in the hematology service at the Botucatu Medical School (UNESP) that monitors patients with hemoglobinopathies, including children with SCA. In both the blood center and in a TCD clinic, patients are examined using the Stroke Prevention Trial in Sickle Cell Anemia (STOP) protocol [16]. This study was approved by the Ethics Committee of the Research of the Botucatu Medical School (reference number: 2,492,335). Parents of the participants signed the informed consent form, while children verbally agreed to the assent form.

### Variables

The outcomes were TAMMV values and episodes of sickle cell crises that occurred during the follow-up period. The following covariates were considered: sex, age, genotype, prescriptions for hydroxyurea, prescriptions for prophylactic transfusions, hemoglobin levels, leukocyte numbers, and platelet counts.

### Data sources and measurement

Clinical, radiological, and laboratory data were extracted from medical records. The flow velocities of the cerebral arteries were evaluated from the files stored in the TCD device the Doppler Box Model, manufactured by Deutschen Wasserball-Liga in Germany. Episodes of sickle cell crises were defined by the attending physician at the time of the event, utilizing data from medical records. Descriptions of the types of crises assessed are shown in Additional File 3. Prescriptions for blood transfusions and hydroxyurea were evaluated, regardless of whether they were prescribed for stroke prophylaxis or for other clinical indications.

### Evaluation protocol with TCD

Examinations were performed with a Doppler device, the TCD Doppler Box from Deutschen Wasserball-Liga (Singen, Germany), using a 2-MHz probe and QL Doppler software version 3.3. The test was performed on Monday mornings, with baseline hemoglobin routinely collected prior to TCD examination. Blood pressure, axillary temperature, and heart rate were also measured prior to the examination. If the patient had a fever, the examination was not performed, as arterial flow velocities could be altered. Biparietal diameter measurements were performed to assess the ideal positions of the evaluated cerebral arteries.

The children were placed in a supine position with parents and/or companions in attendance. The procedure was initiated with a middle transtemporal window to evaluate the velocities of the proximal branches of the middle, anterior, and posterior cerebral arteries. The entire length of the arteries was evaluated at 2 mm intervals, and the flow velocities were recorded using at least two speeds, or more in cases of stenosis. The vertebral and basilar arteries were evaluated through the transforaminal window. The ophthalmic window was only used to evaluate the flow in the ophthalmic arteries and the carotid siphon, if the examination suggested proximal stenosis of the internal carotid artery.

### The STOP protocol

In Brazil, the Decree No. 473/2013 of the Ministry of Health established a protocol for the use of TCD as an outpatient procedure for the prevention of stroke in patients with sickle cell disease. Patients were followed up according to this screening protocol with TCD [11]. The protocol is presented in Table 1.

**Table 1:** Follow-up TCD Protocol for Stroke Prevention in 2 —6 Year Old SCA Patients

Transcranial Doppler Result	TAMMV (cm/s) in Middle Cerebral Artery	Periodicity of Examination
Absence of window	–-	Use another imaging method to analyze the stroke risk
Technical difficulty due to lack of cooperation	–-	Repeat every three months, if possible, by another examiner
Low TAMMV	Less than 70	Repeat after one month, if you continue to use another imaging method to analyze the stroke risk
Normal	Less than 170	Repeat annually
Low conditional	Between 170 and 184	Repeat every three months. In the case of subsequent normal results, the normal group's conduct should be adopted
High conditional	Between 185 and 199	Repeat monthly. In cases of unchanged examinations, it is recommended to repeat every three months. In cases of two atypical examinations, it is recommended to discuss the stroke risk and to consider a chronic transfusion regimen
Abnormal	Greater than or equal to 200— 219	Repeat monthly. If the value remains > 200, it is recommended to discuss the risk of stroke and consider a chronic transfusion regimen. If the result decreases to 170–199, repetition in one month is recommended, if high conditional (between 185 and 199); or in 6 months, if low conditional (between 170 and 184). If the result is normalized (< 170), an annual repetition is recommended
	Greater than or equal to 220	Discuss imminent stroke risk and consider a chronic transfusion regimen

*TCD* Transcranial Doppler, *TAMMV* Time average mean maximum velocity, *SCA* Sickle cell anemia. Adapted from Ordinance No. 473, of April 26, 2013, Ministry of Health

Blood transfusion is performed monthly to maintain an HbS < 30% of the total hemoglobin, a hemoglobin level of 12 g/dL (preferably approximately 10 g/dL), and a hematocrit of approximately 36%. The transfusion protocol is maintained until the patient reaches 16 years of age. Certain patients with normalized TAMMV values after transfusions, who had difficulties or complications following the monthly treatment (such as an inability to attend hospital for treatment), had their treatment changed to hydroxyurea. Additionally, some patients used hydroxyurea after peripheral vaso-occlusive episodes and/or acute chest syndrome, as assessed by Doppler.

### Statistical analysis

Categorical variables are presented as absolute numbers and proportions. Continuous variables are presented as means and standard deviations when they had normal distributions or as medians and interquartile intervals when the distribution was asymmetric. Evaluation of normality of the distribution of continuous variables was based on analysis of their histograms and quantile–quantile plots [17].

We used a shared frailty survival model with a gamma distribution, in which individuals were the random effects component of the model [12]. The decision to use this approach for regression analyses was based on the following arguments. First, the main outcome of interest was the occurrence of recurrent events (i.e., vaso-occlusive syndromes). Second, because TCD was performed on several occasions during follow-up of the participants, a statistical model that allowed for time-varying variables and covariables was required. Survival analyses were performed using simple frailty models, in which each predictor variable was analyzed separately in relation to the occurrence of a sickling crisis, with time measured in days.

Additionally, we performed multivariable analyses in which frailty models involving the same outcome variable and one of the variables related to the TCD results were adjusted for the following covariates: sex, age, genotype, hydroxyurea prescription, prophylactic transfusion prescription, hemoglobin levels, number of leukocytes, and number of platelets in peripheral blood samples. All analyses were performed using the software R (version 3.6.2), (R Core Team 2019); a two-tailed alpha value of 0.05 was adopted for statistical significance.

## Results

### Demographic characteristics

Thirty-seven children who underwent TCD examination were followed up. Table 2 presents the demographic data, genotypes, age at the initiation of treatment, number of TCD examinations performed, and follow-up time for the included patients. Patients with homozygosity for HbS constituted 50% of the sample, with thalassemia co-occurrence the second most common genotype. Four patients received prophylactic blood transfusions and two received blood transfusions at the beginning of the evaluation.

**Table 2:** Clinical/Demographic Characteristics of 37 Children with SCA Assessed with TCD

**Variables**	
**Gender, N (%)**	
Male	28 (75.0%)
Female	9 (25.0%)
**Age, N (%)**	
Median (Q1, Q3)	4.00 (2.0–10.0)
**Genotype N (%),**	
SS	19 (51.3%)
Sβ	8 (21.6%)
Sα	1 (2.7%)
FS	5 (13.5%)
SC	4 (10.8%)
**Years of follow-up**	
Median (Q1, Q3)	6.00 (5.00–7.00)
**No. TCD performed**	
Median (Q1, Q3)	4.00 (2.00–5.00)
**TCD screening, N (%)**	
Conditional	8 (21.6%)
Altered	1 (2.7%)
**Treatment**	
Hydroxyurea	14 (37.8%)
Prophylactic transfusion	4 (11.8%)

*SCD* Sickle cell disease, *TCD* Transcranial Doppler, SS: Homozygous for HbS; Sβ: Heterozygosity for HbS and beta thalassemia; SC: heterozygosity for HbS and HbC; FS: heterozygosity for HbS and persistence of fetal hemoglobin; Sα: heterozygosity for HbS and alpha thalassemia; Q1: First quartile; Q3: Third quartile; *HCFMB* Hospital das Clínicas, Faculty of Medicine of Botucatu. The results are presented as numbers and percentages or medians and percentiles

### Frequency distribution of vaso-occlusive syndromes

Acute chest syndrome was the most common form of vaso-occlusive syndrome (42.9%). Crises secondary to infectious processes were responsible for another third of the total number. While there were no cases of stroke among the study patients, Table 3 presents the frequencies of the types of sickling crises.

**Table 3:** Frequency distribution of vaso-occlusive syndrome types

Episodes of Sickling Crises	N (%)
Splenic infarction	1 (2.4%)
Bone infarction	5 (11.9%)
By infection	13 (31.0%)
Priapism	1 (2.4%)
Pain syndrome	1 (2.4%)
Splenic sequestration	2 (4.8%)
Hepatic sequestration	1 (2.4%)
Acute chest syndrome	18 (42.9%)
Stroke	0 (0.0%)

### Outcomes analysis

The relationships between the speeds measured by the TCD examinations, pulsatility indices (PI), and the relationship between these variables and the occurrence of sickling syndromes, were studied. All genotypes studied were related to a lower risk of sickling than the SS genotype (homozygosity for hemoglobin S). Univariate analyses findings are illustrated in Table 4. When the velocities were studied as continuous variables, we found an increased risk of sickle cell crises when there was an increase in the following: peak systolic velocity (PSV) [hazard ratio (HR): 1.01; 95% confidence interval (CI95%) 1.00—1.02; *p* = 0.04], end-diastolic velocity (EDV) in the middle cerebral artery (MCA) (HR: 1.02; CI95% 1.01—1.04; *p* = 0.01), and TAMMV in the basilar artery (BA) (HR, 1.02; CI 95% 1.00—1.04; *p* = 0.04).

**Table 4:** Univariate Survival Analyses using a Proportional Hazard Frailty Model for Vaso-occlusive Syndrome Outcome

Predictor Variable	HR	CI 95%	*P*
Doppler TAMMV conclusion			
Normal	-	-	-
Intermediate	1.97	0.64—6.07	0.24
Altered	0.89	0.09—8.84	0.92
Doppler TAMMV conclusion			
Normal	-	-	-
Intermediate or altered	1.67	0.60—4.78	0.33
TAMMV Doppler (continuous variable)	1.01	1.00—1.02	0.06
Pulsatility index	0.21	0.01—4.71	0.33
Doppler PSV (cm/s) MCA	1.01	**1.00**—**1.02**	**0.04**
Doppler EDV (cm/s) MCA	1.02	**1.01**—**1.04**	**0.01**
Doppler TAMMV (cm/s) BA	1.02	**1.00**—**1.04**	**0.04**
Sex	0.73	0.26—2.05	0.55
Age	0.98	0.90—1.08	0.74
Genotype			
SS	-	-	-
Sβ ou Sα	0.27	**0.09**—**0.83**	**0.02**
FS	0.14	**0.03**—**0.64**	**0.01**
SC	0.01	**0.16**—**2.31**	**0.47**
Hydroxyurea	1.77	0.82—3.83	0.15
Prophylactic transfusion	1.04	0.27—4.05	0.95
Hb	**0.49**	**0.38** —**0.65**	** < 0.001**
Ht	**0.78**	**0.71**—**0.85**	** < 0.001**
Leukocytes	**1.1**	**1.05**—**1.15**	** < 0.001**
Platelets	**0.997**	**0.994**—**0.999**	**0.02**
Reticulocytes	**1.14**	**1.06**—**1.23**	** < 0.001**

*TAMMV* Time average mean maximum velocity, *MCA* Middle cerebral artery, *PSV* Peak systolic velocities, *EDV* End-diastolic velocity, *BA* Basilar artery, *Hb* Hemoglobin, *Ht* Hematocrit; SS: Homozygous for HbS; Sβ: Heterozygosity for HbS and beta thalassemia; SC: Heterozygosity for HbS and HbC; FS: Heterozygosis for HbS and persistence of fetal hemoglobin; Sα: Heterozygosity for HbS and alpha thalassemia

Reduction in hemoglobin (HR, 0.49; CI 95% CI: 0.38—0.65; *p* < 0.001), hematocrit (HR: 0.78; CI 95%: CI 0.71—0.85; *p* < 0.001), or platelets (HR: 0.99; CI 95% 0.997—0.999; *p* = 0.02), as well as an elevation of leukocytes (HR: 1.1; CI 95% CI 1.05—1.15; *p* < 0.001) and reticulocytes (HR: 1.14; CI 95% CI: 1.06—1.23; *p* < 0.001) were associated with increased risk of vaso-occlusive syndrome.

Associations between TCD analyzed variables and vaso-occlusive syndromes in patients with SCA are described in Table 5.

**Table 5:** Association between the variables analyzed by TCD and vaso-occlusive syndromes in patients with SCA (*n* = 37)

Variable^a^	HR	CI95%	*p*
TAMMV MCS (cm/s)	1.00	0.99—1.01	0.84
PSV MCA (cm/s)	1.00	0.99—1.01	0.72
EDV MCA (cm/s)	1.02	1.00—1.04	0.08
TAMMV BA (cm/s)	1.00	0.97—1.03	0.85
TAMMV altered	0.46	0.05—4.09	0.48
TAMMV conditional or altered	0.90	0.33—2.43	0.83
Pulsatility Index	0.18	0.01—4.43	0.30

*HR* Hazard ratio, *CI* Confidence interval, *SCA* Sickle cell anemia, TCD Transcranial Doppler, *TAMMV* Time average mean maximum velocity, *MCA* Middle cerebral artery, *PSV* Peak systolic velocity, *EDV* End-diastolic velocity, *BA* Basilar artery; ^a^Multivariable Survival analyses using a shared frailty model adjusted for sex, genotype, hydroxyurea prescription, prophylactic blood transfusion, hemoglobin, leukocytes, and platelets

## Discussion

In our study, the PSV, EDV, and TAMMV were associated with occurrence of sickling crises in children with SCA (after univariate analysis). Importantly, Table 4 results regarding univariate associations between the various flow velocities and the hazard of sickling crises, refers to increases of 1 cm/s in Doppler measurements. A hazard ratio of 1.02 was estimated for EDV in the MCA, and is equivalent, on average, to a 20% increase in the risk of experiencing a sickling crisis. The increase of 10 cm/s in the EDV is highly clinically relevant.

Although multivariate analysis did not show independent associations between TCD parameters and sickling crises, univariate analyses indicated that PSV, EDV, and TAMMV may be considered markers of sickling crisis risk. PSV has been suggested as the most sensitive variable for stenosis diagnosis via magnetic resonance imaging of cerebral vessels [18], and the PSV and EDV present a collinear relationship with variations in mean flow velocities [19]. In our study, the PSV and EDV were more sensitive than average flow velocities for diagnosis of vaso-occlusive syndromes.

Flow velocities in cerebral arteries measured by TCD show an inverse relationship with hemoglobin and hematocrit levels [19]. Low hematocrit and hemoglobin levels are associated with low blood viscosity [20]. They are also associated with an increased risk of stroke, kidney disease, pulmonary vasculopathy, and mortality [21]. The pathophysiology of SCA complications, mainly vaso-occlusive syndromes, is closely related to hemolysis, vasculopathy, and chronic anemia [22]. Although TAMMV was used in the STOP protocol (as there is a collinear relationship between PSV and EDV), we thought that these variables could generate additional data and allow us to evaluate any correlations with more severe anemia or complications of SCA. Elevated levels of PSV, EDV, and TAMMV are markers of “hemodynamic stress,” as is TAMMV evaluated in the MCA or ICA.

SCA is the main hemoglobinopathy [3] affecting populations with greater socioeconomic vulnerabilities [23] and a reduced quality of life [24, 25]. Follow-up methods that can predict a higher risk of these complications are essential. TCD is an inexpensive and non-invasive method that has previously been incorporated into the follow-up of patients with sickle cell disease. Increased flow velocities assessed by TCD were related to high risk of stroke. With implementation of a serial transfusion protocol guided by these velocities, it is possible to reduce the risk of complication by 90% [8].

The flow velocities measured by TCD in the cerebral arteries showed an inverse relationship with hemoglobin and hematocrit levels [26]. They were also associated with increased risk of stroke, kidney disease, pulmonary vasculopathy, and mortality [11]. The pathophysiology of SCA complications, especially vessel occlusive syndromes, is closely related to chronic hemolysis, vasculopathy, and anemia [20, 27].

In our study, we found only a small number of patients with abnormal TCD examination findings which may have impaired associations. Only one patient had a TAMMV of > 200 cm/s, and we did not observe stroke occurrence in any of our patients during the follow-up period. These data differ from those reported by Adams et al. [8]; however, they corroborate the results from other authors with cohorts of TCD monitored patients [28, 29]. Additionally, a significant number of patients used a prophylactic therapy such as hydroxyurea or blood transfusions. These treatments relate to a reduction in both cerebral flow velocities [30–32] and complications from sickling syndromes [6, 33]. Unexpectedly, there was no association between hydroxyurea use or prophylactic blood transfusions and reduction in sickling crises.

PI analysis did not show a relationship with the studied outcome variables. This variable reflects vascular resistance and tends to change with age, possibly contributing to the results in ours and other cohort studies [34]. Older patients may show greater changes in this variable.

Interestingly, no patients were diagnosed with stroke during our study period. Despite the small sample number of patients, cerebrovascular events were estimated in up to 11% of patients [7]. These data show the efficiency of current treatments and the TCD follow-up protocol, despite the difficulties faced by our health system. The most common complications were acute chest syndrome and sickling crises due to infection. These complications are associated with high morbidity and mortality [1, 20, 35]. Treatments, such as blood transfusions and hydroxyurea, reduce acute chest crises and vaso-occlusive syndromes [36, 37]. Thus, more rigorous follow-up with TCD could benefit patients by initiating these treatments earlier.

Regarding genotypes, homozygosity for HbS presented an increased risk of crises when compared to all other evaluated subtypes. This corroborates previous findings and shows a greater severity of the SS genotype [2]; it is also related to the higher speeds in TCD examination in other studies [10, 32]. Unfortunately, many patients do not begin follow-up, begin follow-up late, or abandon follow-up altogether. In a cohort of patients from São Paulo, the mean age of follow-up onset was 9 years [29]; this was much greater than in our study where the mean age of follow-up onset was 4 years. Reeves et al. reported TCD screening in only 30% of patients [38]. In our cohort, patients began follow-up at 4 years of age and had an average follow-up of 6 years, with four examinations. That is, the examinations began late, and the patients underwent less than one examination per year, leaving follow-up prematurely.

TCD screening for patients with SCA is included in the American National Institute of Health and British National Health System guidelines, and there are many experiences in other countries and populations that show clear benefit and feasibility [39–41]. However, implementation of these protocols is far from ideal. Overburdening children and families with appointments, not including TCD in routine hematological visits, and poor understanding of TCD usefulness for prevention of serious complications are usually identified as barriers to treatment [40]. Education for staff and parents and same day TCD examinations and consultations are suitable approaches to overcome these problems.

This study had a few limitations. This was a retrospective cohort study with few patients from a single center. However, this was a real-life study, with patients followed according to the not-always-optimal conditions found in the public health system. Awareness and dissemination of TCD technology is beneficial to patients with SCA. The examination is inexpensive, non-invasive, and directly related to the pathophysiology of the disease. New studies using this method can add great benefit to patient treatment without associated morbidity or additional burdens on the health care system.

## Conclusions

Our results highlight peak systolic and end-diastolic velocities, measured in the MCA and the TAMMV of the BA. These can be used as markers of risk for occurrence of sickling crises in SCA. The examination is inexpensive, non-invasive, and directly related to disease pathology. Future studies should confirm our findings and examine the use of TCD to prevent sickling crises.

## Supplementary Information

**Additional file 1.** Clinical Manifestations of Sickle Cell Anemia.

**Additional file 2.** Pathophysiology of Sickle Cell Anemia.

**Additional file 3.** Definition of Types of Sickling Crises.

## Data Availability

The datasets generated and/or analyzed during the current study are not publicly available to maintain the anonymity of participants but are available from the corresponding author on reasonable request.

## Abbreviations

BA: Basilar artery
EDV: End-diastolic velocity
HCFMB: Hospital das Clinics, Faculty of Medicine of Botucatu
MCA: Middle cerebral artery
PI: Pulsatility indices
PSV: Peak systolic velocity
SCA: Sickle cell anemia
SCCRIP: Sickle Cell Clinical Research and Intervention Program
STOP: Syndrome during the Stroke Prevention
TAMMV: Time average mean maximum velocity
TCD: Transcranial Doppler

## References

[1] Ware RE, de Montalembert M, Tshilolo L, Abboud MR (2017). Sickle cell disease. Lancet.

[2] Piel FB, Steinberg MH, Rees DC (2017). Sickle Cell Disease. N Engl J Med.

[3] Elguero E, Délicat-Loembet LM, Rougeron V, Arnathau C, Roche B, Becquart P (2015). Malaria continues to select for sickle cell trait in Central Africa. Proc Natl Acad Sci U S A.

[4] Du S, Lin C, Tao YX (2019). Updated mechanisms underlying sickle cell disease-associated pain. Neurosci Lett.

[5] Pinto VM, Balocco M, Quintino S, Forni GL (2019). Sickle cell disease: A review for the internist. Intern Emerg Med.

[6] Ware RE, Davis BR, Schultz WH, Brown RC, Aygun B, Sarnaik S (2016). Hydroxycarbamide versus chronic transfusion for maintenance of transcranial doppler flow velocities in children with sickle cell anaemia-TCD With Transfusions Changing to Hydroxyurea (TWiTCH): A multicentre, open-label, phase 3, non-inferiority trial. Lancet.

[7] Qureshi N, Lubin B, Walters MC (2006). The prevention and management of stroke in sickle cell anaemia. Expert Opin Biol Ther.

[8] Adams RJ, McKie VC, Hsu L, Files B, Vichinsky E, Pegelow C (1998). Prevention of a First Stroke by Transfusions in Children with Sickle Cell Anemia and Abnormal Results on Transcranial Doppler Ultrasonography. N Engl J Med.

[9] Enninful-Eghan H, Moore RH, Ichord R, Smith-Whitley K, Kwiatkowski JL (2010). Transcranial Doppler ultrasonography and prophylactic transfusion program is effective in preventing overt stroke in children with sickle cell disease. J Pediatr.

[10] Hokazono M, Silva GS, Silva EM, Braga JA (2011). Results from transcranial Doppler examination on children and adolescents with sickle cell disease and correlation between the time-averaged maximum mean velocity and hematological characteristics: A cross-sectional analytical study. Sao Paulo Med J.

[11] Ataga KI, Gordeuk VR, Agodoa I, Colby JA, Gittings K, Allen IE (2020). Low hemoglobin increases risk for cerebrovascular disease, kidney disease, pulmonary vasculopathy, and mortality in sickle cell disease: A systematic literature review and meta-analysis. PLoS ONE.

[12] Inusa BPD, Sainati L, MacMahon C, Colombatti R, Casale M, Perrotta S (2019). An educational study promoting the delivery of transcranial Doppler ultrasound screening in paediatric sickle cell disease: A European multi-centre perspective. J Clin Med.

[13] Schlenz AM, Phillips S, Mueller M, Melvin C, Adams RJ, Kanter J (2020). Practice patterns for stroke prevention using transcranial Doppler in sickle cell anemia: DISPLACE Consortium. Pediatr Blood Cancer.

[14] Estepp JH, Cong Z, Agodoa I, Kang G, Ding J, McCarville MB (2021). What drives transcranial Doppler velocity improvement in paediatric sickle cell anaemia: Analysis from the Sickle Cell Clinical Research and Intervention Program (SCCRIP) longitudinal cohort study. Br J Haematol.

[15] Salama K, Rady R, Hashem RH, El-Ghamrawy M (2020). Transcranial Doppler velocities among sickle cell disease patients in steady state. Hemoglobin.

[16] Novelli EM, Gladwin MT (2016). Crises in sickle cell disease. Chest.

[17] Field A, Miles J, Field Z (2012). Discovering statistics using R.

[18] Lanzkron S, Patrick Carroll C, Haywood C (2013). Mortality rates and age at death from sickle cell disease: U.S., 1979–2005. Public Health Rep.

[19] Uwaezuoke SN, Ayuk AC, Ndu IK, Eneh CI, Mbanefo NR, Ezenwosu OU (2018). Vaso-occlusive crisis in sickle cell disease: Current paradigm on pain management. J Pain Res.

[20] Sundd P, Gladwin MT, Novelli EM (2019). Pathophysiology of sickle cell disease. Annu Rev Pathol Mech Dis.

[21] Brousseau DC, Owens PL, Mosso AL, Panepinto JA, Steiner CA (2010). Acute care utilization and rehospitalizations for sickle cell disease. JAMA.

[22] Smith WR, Penberthy LT, Bovbjerg VE, McClish DK, Roberts JD, Dahman B (2008). Daily assessment of pain in adults with sickle cell disease. Ann Intern Med.

[23] Fernandes TA, Medeiros TM, Alves JJ, Bezerra CM, Fernandes JV, Serafim ÉS (2015). Socioeconomic and demographic characteristics of sickle cell disease patients from a low-income region of northeastern Brazil. Rev Bras Hematol Hemoter.

[24] Manci EA, Culberson DE, Yang YM, Gardner TM, Powell R, Haynes J (2003). Causes of death in sickle cell disease: An autopsy study. Br J Haematol.

[25] Serjeant GR (2013). The natural history of sickle cell disease. Cold Spring Harb Perspect Med.

[26] De Melo HA, Barreto-Filho JAS, Do Prado RCP, Cipolotti R (2008). Transcranial Doppler in sickle cell anaemia: Evaluation of brain blood flow parameters in children of Aracaju. Northeast - Brazil Arq Neuro Psiquiatr.

[27] Kato GJ, Steinberg MH, Gladwin MT (2017). Intravascular hemolysis and the pathophysiology of sickle cell disease. J Clin Invest.

[28] Asbeutah AM, AlMajran AA, Adekile A (2019). Pattern of cerebral blood flow and the interrelationship of vascular parameters of transcranial Doppler imaging in children with sickle cell disease. J Clin Ultrasound.

[29] Rodrigues DLG, Adegoke SA, Campos RS de SM, Braga JAP, Figueiredo MS, Silva GS (2017). Patients with sickle cell disease are frequently excluded from the benefits of transcranial Doppler screening for the risk of stroke despite extensive and compelling evidence. Arq Neuro Psiquiatr.

[30] Hankins JS, McCarville MB, Rankine-Mullings A, Reid ME, Lobo CL, Moura PG (2015). Prevention of conversion to abnormal transcranial Doppler with hydroxyurea in sickle cell anemia: A Phase III international randomized clinical trial. Am J Hematol.

[31] Lagunju IO, Brown BJ, Sodeinde O (2015). Hydroxyurea lowers transcranial Doppler flow velocities in children with sickle cell anaemia in a Nigerian cohort. Pediatr Blood Cancer.

[32] Adegoke SA, Macedo-Campos RS, Braga JAP, Figueiredo MS, Silva GS (2018). Changes in transcranial Doppler flow velocities in children with sickle cell disease: The impact of hydroxyurea therapy. J Stroke Cerebrovasc Dis.

[33] Wang WC, Ware RE, Miller ST, Iyer RV, Casella JF, Minniti CP (2011). Hydroxycarbamide in very young children with sickle-cell anaemia: A multicentre, randomised, controlled trial (BABY HUG). Lancet.

[34] Macedo-Campos RS, Adegoke SA, Figueiredo MS, Braga JAP, Silva GS (2018). Cerebral vasoreactivity in children with sickle cell disease: A transcranial Doppler study. J Stroke Cerebrovasc Dis.

[35] Ochocinski D, Dalal M, Black LV, Carr S, Lew J, Sullivan K (2020). Life-threatening infectious complications in sickle cell disease: A concise narrative review. Front Pediatr.

[36] Miller ST, Wright E, Abboud M, Berman B, Files B, Scher CD (2001). Impact of chronic transfusion on incidence of pain and acute chest syndrome during the Stroke Prevention Trial (STOP) in sickle-cell anemia. J Pediatr.

[37] Charache S, Terrin ML, Moore RD, Dover GJ, Barton FB, Eckert SV (1995). Effect of hydroxyurea on the frequency of painful crises in sickle cell anemia. Investigators of the Multicenter Study of Hydroxyurea in Sickle Cell Anemia. N Engl J Med..

[38] Reeves SL, Fullerton HJ, Cohn LM, Dombkowski KJ, Boulton ML, Braun TM (2016). Missed opportunities for transcranial Doppler screening among children with sickle cell disease. Clin Pediatr (Phila).

[39] Leite AC, Oliveira RV, Moura PG, Silva CM, Lobo C (2012). Abnormal transcranial Doppler ultrasonography in children with sickle cell disease. Rev Bras Hematol Hemoter.

[40] Colombatti R, Meneghetti G, Ermani M, Pierobon M, Sainati L (2009). Primary stroke prevention for sickle cell disease in north-east Italy: The role of ethnic issues in establishing a transcranial Doppler screening program. Ital J Pediatr.

[41] Ghafuri DL, Covert Greene BC, Musa B, Gambo A, Sani A, Abdullahi S (2021). Capacity building for primary stroke prevention teams in children living with sickle cell anemia in Africa. Pediatr Neurol.

