# Supplementary figures and images for: Transcranial doppler as screening method for sickling crises in children with sickle cell anemia: a latin America cohort study
Source: BMC Pediatr. 2022 Jun 27;22:368. doi: 10.1186/s12887-022-03429-5 (PMC9235247; doi:10.1186/s12887-022-03429-5)

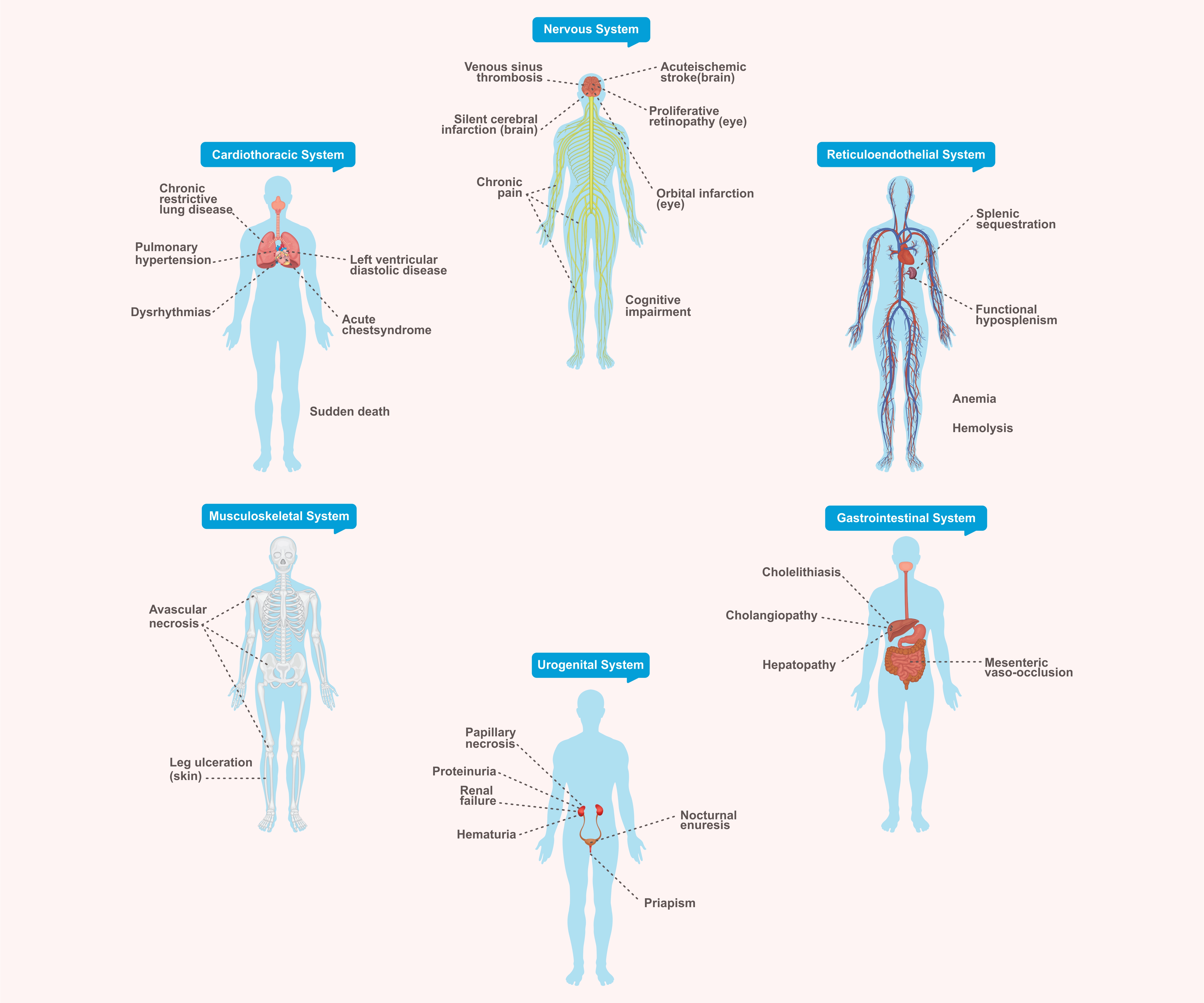

Supplement: Supplementary file 1 — Additional file 1. Clinical Manifestations of Sickle Cell Anemia. [file 12887_2022_3429_MOESM1_ESM.jpg]

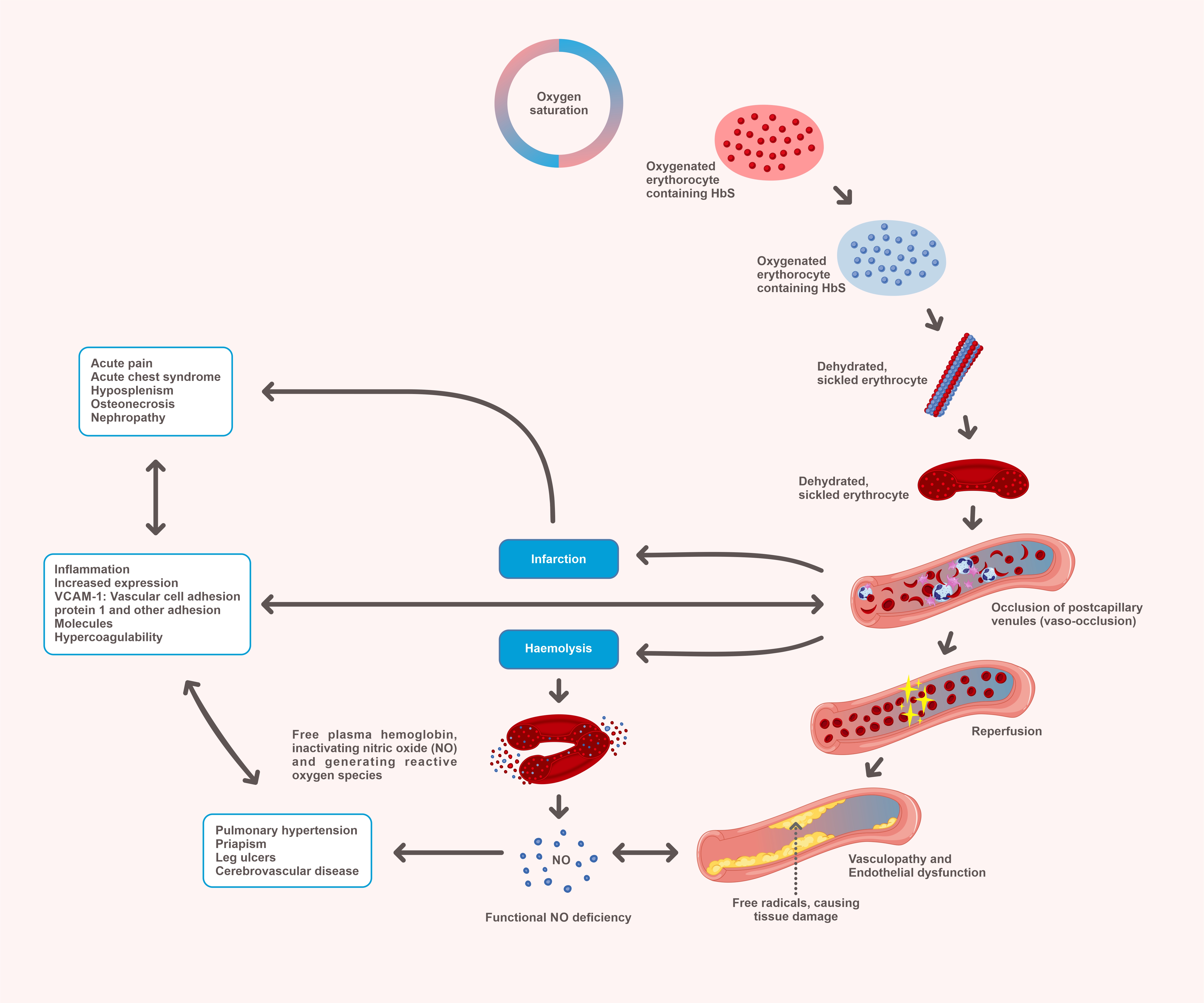

Supplement: Supplementary file 2 — Additional file 2. Pathophysiology of Sickle Cell Anemia. [file 12887_2022_3429_MOESM2_ESM.jpg]
