# Supplementary material for: Transcranial doppler as screening method for sickling crises in children with sickle cell anemia: a latin America cohort study
Source: BMC Pediatr. 2022 Jun 27;22:368. doi: 10.1186/s12887-022-03429-5 (PMC9235247; doi:10.1186/s12887-022-03429-5)
Supplement: Supplementary file 3 — Additional file 3. Definition of Types of Sickling Crises. [file 12887_2022_3429_MOESM3_ESM.docx]

**Supplemental file 3.** Definition of the types of sickling crises

| *Crisis Type* | *Definitions* |
| --- | --- |
| Stroke | defined as an image compatible with ischemic insult on tomography or with magnetic resonance of the brain, or clinical observations compatible with sudden neurological deficit secondary to ischemia |
| Splenic infarction | presence of a splenic ischemic area in image examination (computed tomography or ultrasound) |
| Pain syndrome | defined as the presence of acute onset of pain due to recent vascular occlusion due to sickling |
| Bone infarction | characterized by an ischemic/occlusive bone lesion, defined by radiography (in the case of aseptic necrosis of the femoral head) or bone scintigraphy |
| Priapism | painful penile erection, which occured independently of sexual desire, lasting more than two hours, without leading to ejaculation, caused by insufficient drainage of the blood that fills the corpora cavernosa |
| Splenic sequestration | reduction in hemoglobin concentration equal to or greater than 2g / dl compared to the patient's baseline value, increased erythropoiesis and spleen/liver dimensions |
| Hepatic sequestration | right upper quadrant pain, hepatomegaly, drop in hematocrit, increased reticulocytes and little change in liver enzymes |
| Acute chest syndrome | defined as a new infiltrate detected on chest radiography in a patient with acute respiratory syndrome |
